# Among Early Appearing Non-Motor Signs of Parkinson’s Disease, Alteration of Olfaction but Not Electroencephalographic Spectrum Correlates with Motor Function

**DOI:** 10.3389/fneur.2017.00545

**Published:** 2017-10-20

**Authors:** Vitalii V. Cozac, Bianca Auschra, Menorca Chaturvedi, Ute Gschwandtner, Florian Hatz, Antonia Meyer, Antje Welge-Lüssen, Peter Fuhr

**Affiliations:** ^1^Department of Neurology and Neurophysiology, University Hospital of Basel, Basel, Switzerland; ^2^Department of Mathematics and Computer Science, University of Basel, Basel, Switzerland; ^3^Department of Otorhinolaryngology, University Hospital of Basel, Basel, Switzerland

**Keywords:** olfaction, sniffing test, Parkinson’s disease, electroencephalographic, Unified Parkinson’s Disease Rating Scale-III

## Abstract

Olfactory decline is a frequent and early non-motor symptom in Parkinson’s disease (PD), which is increasingly used for diagnostic purposes. Another early appearing sign of PD consists in electroencephalographic (EEG) alterations. The combination of olfactory and EEG assessment may improve the identification of patients with early stages of PD. We hypothesized that olfactory capacity would be correlated with EEG alterations and motor and cognitive impairment in PD patients. To the best of our knowledge, the mutual influence of both markers of PD—olfactory decrease and EEG changes—was not studied before. We assessed the function of odor identification using olfactory “Screening 12 Test” (“Sniffin’ Sticks^®^”), between two samples: patients with PD and healthy controls (HC). We analyzed correlations between the olfactory function and demographical parameters, Unified Parkinson’s Disease Rating Scale (UPDRS-III), cognitive task performance, and spectral alpha/theta ratio (α/θ). In addition, we used receiver operating characteristic-curve analysis to check the classification capacity (PD vs HC) of olfactory function, α/θ, and a combined marker (olfaction and α/θ). Olfactory capacity was significantly decreased in PD patients, and correlated with age, disease duration, UPDRS-III, and with items of UPDRS-III related to gait and axial rigidity. In HC, olfaction correlated with age only. No correlation with α/θ was identified in both samples. Combined marker showed the largest area under the curve. In addition to EEG, the assessment of olfactory function may be a useful tool in the early characterization and follow-up of PD.

## Introduction

The decline of olfaction (hyposmia) is a feature of an early stage of the Parkinson’s disease (PD), which may be useful as a premotor biomarker in PD (1–5). In some cases, the olfactory decline is one of the cardinal signs of PD, thus, in a recently suggested classification of the non-motor subtypes of PD, “Park weight subtype” concludes phenotypes with olfactory impairment and risk of dyskinesia (6). In addition, the olfactory decline may be helpful in differential diagnosis of PD (7). Hyposmia has a high diagnostic accuracy, in comparison with other neurodegenerations; in PD, the olfactory disturbance is much more severe (8–10). However, in early stages of PD, the application of olfactory assessment as a single screening tool is not reasonable, and additional markers are required for a precise diagnosis (11).

Another early appearing sign of PD consists in electroencephalographic (EEG) alterations (12). Quantitative spectral analysis of the EEG is another method to differentiate PD patients from healthy controls (HC), showing that an increase of the spectral power of “slow” (<8 Hz) waves is specific for PD (13–15).

Thus, we assumed that olfactory function in PD correlates with spectral EEG parameters and that the combination of smell identification testing and EEG may increase the precision of PD identification. To the best of our knowledge, the mutual influence of both markers of PD—olfactory decrease and EEG changes—was not studied before.

In this study, we aimed: (a) to compare olfactory function between groups of PD patients and HC using Sniffin’ Sticks; (b) to check the correlations between the olfaction capacity [assessed with sniffing score (SnSc)], EEG (alpha/theta power ratio), and motor features; and (c) to check the added value of SnSc to EEG, to classify PD vs controls.

## Materials and Methods

We performed a cross-sectional retrospective study of two samples of participants: PD patients and HC. These participants were selected from a study database from the Hospital of the University of Basel (Switzerland) according to the availability of the results of smell identification testing. The respective study is an ongoing observational cohort investigation, focused on the EEG and genetic markers of cognitive outcomes in PD; the details of this study are provided elsewhere (16). HC were recruited through advertisements in local newspapers and were screened by neuropsychologist and neurologist to exclude presence of chronic neurological diseases (e.g., PD, multiple sclerosis, and history of stroke). The PD sample comprised 54 patients (median age 68 years, males 69%), and the HC sample comprised 21 participants (median age 67 years, males 67%). The sample size to detect intergroup difference (PD vs HC) is capable of detecting an effect size as low as 0.73 with a statistical power of 80% at a 5% significance level. Sample size of PD patients (*n* = 54) is capable to detect a correlation coefficient as low as 0.37 with a statistical power of 80% at a 5% significance level; and sample size of HC participants (*n* = 21) is capable to detect a correlation coefficient as low as 0.57 with a statistical power of 80% at a 5% significance level.

In both samples, we analyzed the following baseline tools: Mini-Mental State Examination (MMSE), five neuropsychological tests, EEG, and olfactory “Screening 12 Test” (“Sniffin’ Sticks^®^,” commercially available, Burghart Messtechnik GmbH, Wedel, Germany). Only the PD sample was assessed with Unified Parkinson’s Disease Rating Scale (UPDRS). Levodopa equivalent of the daily dose (LEDD) of the antiparkinsonian medication was calculated (17). All participants provided written informed consent to the processing of personal data within the study, which was approved by the local ethics committee (*Ethikkommission beider Basel, letters No. 135/11 and 294/13*).

### Assessment of the Olfactory Function

The olfactory function was assessed using the Sniffin Sticks Screening 12 Test, which consists of 12 felt-tip pens filled with an odorant, e.g., orange, coffee, and fish (18). Removal of the cap releases the odor. The type of the odorant is coded and is not known to the examinee. The pen is held approximately 2 cm in front of the examinee’s nostrils, and the examinee receives a verbal command to inhale the odor with both nostrils for 2 s. Then, the examinee is given a card with four variants of odor (including the correct one), and—in a forced choice paradigm—is asked to select the correct odor. The number of correctly identified odorants is summed up to calculate the “SnSc” ranging from 0 to 12.

### EEG Processing

We recorded continuous EEG with 214 active electrodes in each participant, in a relaxed eyes-closed state. The electrode located at *C*_Z_ was used as a reference (Net Station 300; Electrical Geodesics, Inc). All recordings were processed with “TAPEEG” toolbox (19). The sampling rate was set at 1,000 Hz; oscillations were filtered with 2,500 order least-square filter with band-pass 0.5–70 Hz and notch 50 Hz. Detection and removal of artifacts (e.g., eye blinks) were fully automated, by an independent component analysis. Channels with bad activations were automatically detected and interpolated by spherical spline method. Global relative median power was calculated in frequency ranges: θ (4–8 Hz) and α (8–13 Hz). Alpha/theta ratio (α/θ) was subsequently calculated. In other words, α/θ is an indicator of EEG slowing, the smaller the ratio, the slower the EEG.

### Cognitive Tests

We used the following five tests: Wisconsin Card Sorting Test: correct categories (WCST), Trail Making Test time for part A (TMTA), Test of Attentional Performance—Working Memory (2-back task): omissions (TAPWMO), Semantic verbal fluency test: correct answers (SVFC), and Phonemic verbal fluency: correct answers (PVFC). Test variables were normalized with reference to a normative database of 604 HC from the Memory Clinic, Felix Platter Hospital of Basel, Switzerland (20).

### Statistics

Statistical calculations were performed with R tool for statistical calculations (21). We used corrected Wilcoxon and chi-squared tests to compare variables between the samples. Spearman rank correlation test was applied to check the relation of SnSc with the following parameters: age, sex, disease duration (since the first diagnosis), years of education, MMSE, LEDD, α/θ, UPDRS-III, WCST, TMTA, TAPWMO, SVFC, and PVFC.

We applied receiver operating characteristic (ROC) curves to analyze the classification value of the following variables: SnSc, α/θ, and a combined score (as sum of SnSc and α/θ). For ROC-curve analyses, PD and HC samples were merged, and the presence of PD was used as an outcome.

Bonferroni correction for multiple testing was applied. The level of statistical significance was set at 0.05. Power analysis was performed according to Cohen (22).

## Results

The samples are shown in Table 1. In comparison with HC, in PD patients, the following parameters were significantly decreased: SnSc, WCST, TMTA, SVFT, and α/θ.

**Table 1:** Comparison of the PD sample with HC sample.

Parameter	PD, *n* = 54	HC, *n* = 21	*p*-Value (95% conf. int.)
Males, *n* (%)	37 (69)	14 (67)	ns
Age, years	68 (45, 85)	67 (57, 78)	ns
Years of education	15 (9, 20)	15 (10, 20)	ns
MMSE	29 (24, 30)	30 (26, 30)	ns
Disease duration, years	3.41 (1, 23)	–	–
UPDRS-III	18 (1, 47)	–	–
LEDD, mg/day	475 (0, 2,950)	–	–
SnSc	5.5 (2, 12)	10 (7, 12)	*p* < 0.001 (−5.0, −2.0)
WCST	−0.54 (−2.13, 3.23)	0.05 (−1.33, 2.17)	*p* < 0.05 (−0.9, −0.1)
SVFC	−0.30 (−2.08, 1.94)	0.11 (−1.42, 2.89)	*p* < 0.05 (−1.2, −0.1)
PVFC	0.22 (−1.95, 2.56)	0.22 (−1.72, 1.96)	ns
TMTA	−0.44 (−3.09, 2.27)	0.57 (−1.11, 3.34)	*p* < 0.01 (−1.5, −0.3)
TAPWMO	0.25 (−2.33, 2.80)	−0.10 (−2.33, 2.32)	ns
α/θ	1.32 (0.24, 5.39)	1.96 (0.79, 7.71)	*p* < 0.05 (−1.0, −0.01)

*Comparison of the PD sample with HC sample. For continuous parameters Wilcoxon test with Bonferroni correction was applied; number of males was compared with Chi-squared test; ns = p > 0.05*.

*PD, Parkinson’s disease; HC, healthy controls; MMSE, Mini-Mental State Examination; UPDRS, Unified Parkinson’s Disease Rating Scale; LEDD, levodopa equivalent of the daily dose; SnSc, sniffing score*.

In PD sample, SnSc correlated with age, disease duration, UPDRS-III, and the following items of UPDRS-III: “Postural stability” (rho = −0.43, *p* < 0.01), “Leg agility, right” (rho = −0.43, *p* < 0.01), “Gait” (rho = −0.29, *p* < 0.05), and “Rigidity neck” (rho = −0.30, *p* < 0.05) (Tables 2 and 3).

**Table 2:** Correlation of SnSc with samples’ characteristics.

Parameter	Rho (PD sample)	Rho (HC sample)
Male sex	−0.05	−0.29
Age, years	−0.70***	−0.42**
Years of education	0.23	0.21
MMSE	0.30	0.30
Disease duration, years	−0.49***	–
UPDRS-III	−0.70**	–
LEDD, mg/day	−0.24	–
WCST	0.02	0.08
SVFC	−0.03	−0.08
PVFC	−0.20	−0.12
TMTA	0.25	−0.10
TAPWMO	0.25	0.10
α/θ	0.23	0.20

*p < 0.05; **p < 0.01; ***p < 0.001.

*PD, Parkinson’s disease; HC, healthy controls; MMSE, Mini-Mental State Examination; UPDRS, Unified Parkinson’s Disease Rating Scale; LEDD, levodopa equivalent of the daily dose; SnSc, sniffing score*.

**Table 3:** Correlation of SnSc with items of UPDRS-III in Parkinson’s disease sample.

Items	Rho
Speech	−0.15
Facial expression	−0.23
Tremor at rest: face	−0.14
Tremor at rest: right upper extremity	−0.12
Tremor at rest: left upper extremity	−0.05
Tremor at rest: right lower extremity	−0.05
Tremor at rest: left lower extremity	−0.01
Action or postural tremor of hands: right	−0.08
Action or postural tremor of hands: left	−0.22
Rigidity: neck	−0.30*
Rigidity: right upper extremity	−0.20
Rigidity: left upper extremity	−0.19
Rigidity: right lower extremity	−0.24
Rigidity: left lower extremity	−0.24
Finger taps: right	−0.05
Finger taps: left	−0.10
Hand movements: right	−0.12
Hand movements: left	−0.12
Rapid alternating movement of hands: right	−0.08
Rapid alternating movement of hands: left	−0.24
Leg agility: right	−0.43***
Leg agility: left	−0.24
Arising from chair	−0.12
Posture	−0.25
Gait	−0.29**
Postural stability	−0.43***
Body bradykinesia and hypokinesia	−0.23

*p < 0.05; **p < 0.01; ***p < 0.001.

*UPDRS, Unified Parkinson’s Disease Rating Scale; SnSc, sniffing score*.

In HC sample, SnSc correlated with age only (rho = −0.62, *p* < 0.05).

No correlation of SnSc with α/θ was identified in both samples.

**Figure 1:**
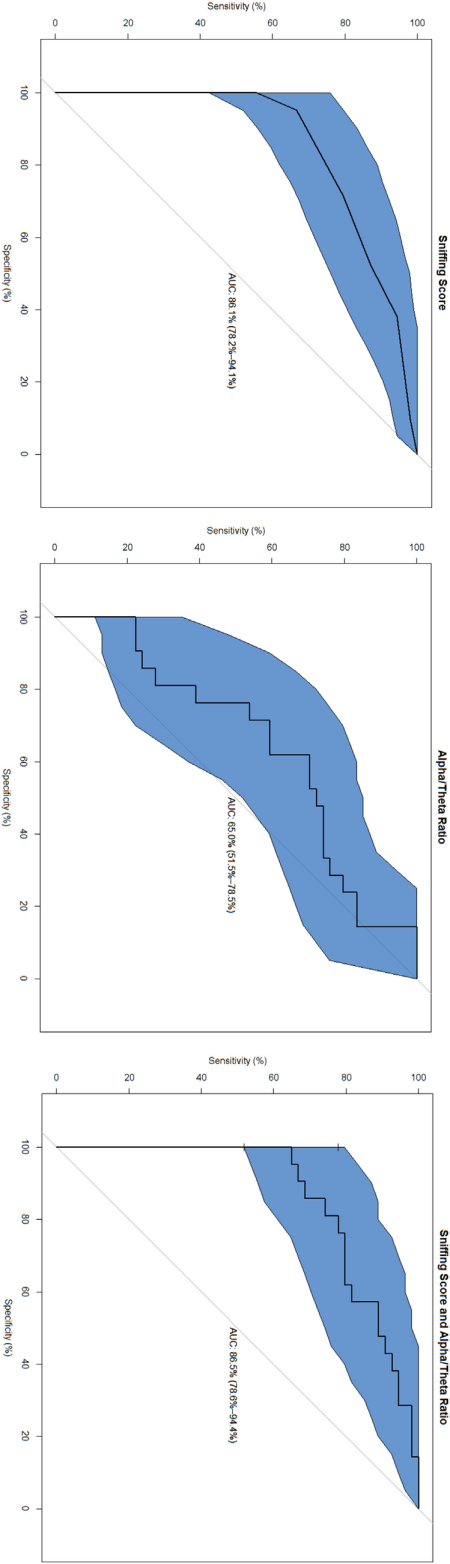
Receiver operating characteristic curves.

The highest area under the curve (AUC) was found in the combined marker (SnSc + α/θ: 86.5%, specificity 100%, sensitivity 64.8%); followed by SnSc (AUC 86.1%, specificity 95.2%, sensitivity 66.7%), and α/θ (AUC 65.0%, specificity 61.9%, sensitivity 70.3%) (Figure 1).

## Discussion

To the best of our knowledge, this is the first study to analyze correlations of olfactory decline with EEG slowing and motor impairment in patients with PD. In our study, we can distinguish three important findings. First, odor identification capacity is significantly lower in PD patients than in HC. The exact mechanism of this olfactory decline in PD is not fully understood. However, the concept of neuropathological staging by Braak et al., and further evolved by Ubeda-Bañon et al., implies a consecutive accumulation of pathological α-synuclein (SNCA) in the olfactory bulb and in other olfactory-eloquent brain regions already in the early stages of the disease (23, 24), subsequently associated with atrophy (25, 26). Studies in European (27) and in Chinese populations (28) of PD patients showed that olfactory impairment is also related to advanced age. As normal aging is also associated with some degree of olfactory decline (25), we assume that at least two factors contributing to olfactory decline are present in patients with PD: “age” and the specific disease process. Nevertheless, the sensitivity and specificity of the evaluation of olfaction to classify PD patients and controls (and some other forms of parkinsonism) is equal or even exceeds that of other biomarkers (26).

Second, the decrease of olfaction correlates with motor impairment in PD, more specifically with gait impairment and axial rigidity, but not tremor. This result corroborates that of a Japanese study in which olfactory function in patients with akinetic-rigid PD was significantly lower than in patients with tremor-dominant and mixed forms (29). Association of olfactory impairment and mobility parameters may be explained by the projections from the olfactory tracts to brain structures (30), involved in movement planning, spatial navigation, and sensorimotor integration: e.g., frontal cortex and cerebellum (31, 32). It is worth to mention that a longitudinal analysis of nine large cohorts of patients with PD showed a correlation of the degree of motor impairment with cognitive decline (33); in that study UPDRS-III was part of a compound cognitive risk score (next to age at onset, MMSE, years of education, sex, depression, and β-glucocerebrosidase mutation). In another longitudinal observation, olfactory decline increased the risk of dementia up to 10 years after PD diagnosis regardless of baseline cognitive function (34). We might suggest that olfactory assessment may contribute to the compound cognitive decline risk score.

Third, we found no association between olfaction and resting-state EEG power spectrum. The likely reason for this fact is that the neurodegeneration occurs in different systems simultaneously but independently, and at a different pace. Alternatively, the sensitivity of methods may differ. Interestingly, a cohort study combining psychophysiological assessment of the olfactory function with olfactory event-related potentials (OERP) found a pattern of fluctuation (mostly decrease) of OERP over time (35). Elicitation of OERP depends on the integrity of the structures conducting impulses from the amygdala to cortical representation areas. The neurodegenerative process causing PD may disturb the connections responsible for OERP as well as those responsible for fast frequencies of the EEG. Since no strong association between alterations of global EEG power spectrum and olfactory deficits can be detected, these measures contain independent information, which in combination may serve as independent factors in statistical models of PD.

Our study has some limitations. First, the “Screening 12 Test” means “forced” selection of one correct odor of four on each of the 12 cards; thus there is a theoretical 25% chance of random selection of the correct answer. In this regard, a larger comprehensive olfactory test battery would allow a more precise identification of the level of hyposmia. Second, the UPDRS-III tool comprises a separate bilateral motor assessment, but the olfactory test which we applied assessed olfaction in both nostrils at the same time. An analysis of the association between sides of olfactory and motor impairment would be interesting. Finally, a selection bias may be present because of the single-site cross-sectional setting of our study. Studies with larger samples and multiple centers could overcome this limitation.

In conclusion, olfactory decrease in PD correlates with motor impairment in lower extremities and gait difficulties, and therefore, the assessment of olfactory function could be a predictor of the loss of independence. EEG slowing is associated with cognitive decline, which is also a predictor of the loss of independence (35–38).

The combination of both tests may constitute a valuable candidate for a powerful prognostic composite biomarker, and longitudinal studies combining both methods are warranted.

## Ethics Statement

All participants provided written informed consent to the processing of personal data within the study, which was approved by the local ethics committee (Ethikkommission beider Basel, letters No. 135/11 and 294/13).

## Author Contributions

VC: conceived and designed the study and was responsible for its execution, wrote the first draft, performed data analyses, contributed core ideas, and was involved in critically revising the manuscript; BA: was responsible for its execution, performed data analyses, and contributed core ideas; MC: performed data analyses and contributed core ideas; UG: conceived and designed the study and was responsible for its execution, performed data analyses, was responsible for the psychiatric and psychological assessments, contributed core ideas, and was involved in critically revising the manuscript; FH: contributed core ideas and was involved in critically revising the manuscript; AM: was responsible for the psychological assessments, contributed core ideas, and was involved in critically revising the manuscript; AW-L: contributed core ideas and was involved in critically revising the manuscript; PF: conceived and designed the study and was responsible for its execution, contributed core ideas, and was involved in critically revising the manuscript.

## Conflict of Interest Statement

The authors declare that the research was conducted in the absence of any commercial or financial relationships that could be construed as a potential conflict of interest.
